# Supplementary material for: Different Metabolites of the Gastric Mucosa between Patients with Current Helicobacter pylori Infection, Past Infection, and No Infection History
Source: Biomedicines. 2022 Feb 26;10(3):556. doi: 10.3390/biomedicines10030556 (PMC8945329; doi:10.3390/biomedicines10030556)
Supplement: Supplementary file 1 [file biomedicines-10-00556-s001.zip › biomedicines-1596989-supplementary.pdf]

Supplementary Materials

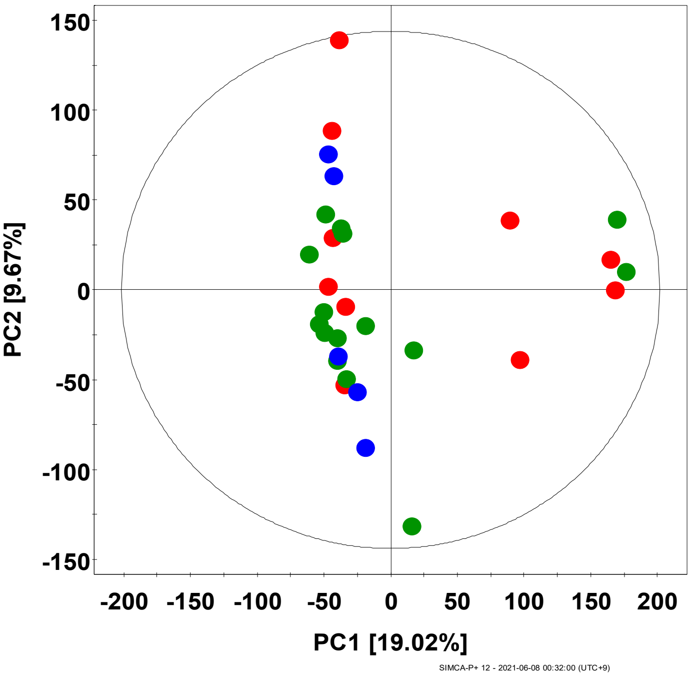

Figure S1. Principal component analysis score plot derived from GC-TOF-MS data of gastric mucosa samples from the three different groups (CI, PI, and NI group). Symbol: current infection (CI, ●), past infection (PI, ●), and no history of infection (NI, ●). To determine the statistical significance, $p < 0.05$ obtained from one-way ANOVA was applied.

No gastritis	Hp-negative gastritis	Hp gastritis	Metabolites	
0.98	1.32	0.74	Pyruvic acid	*Organic acids*
0.80	0.68	1.50	Fumaric acid	
0.77	0.60	1.61	Malic acid	
0.80	0.50	1.66	Citric acid	
1.12	1.03	0.84	Valine	*Amino acids*
1.14	1.06	0.80	Leucine	
1.13	1.04	0.82	Isoleucine	
0.89	0.68	1.41	GABA	
1.02	1.09	0.89	Phenylalanine	
1.02	1.20	0.80	Asparagine	
0.97	1.09	0.95	Histidine	
1.06	1.12	0.83	Tyrosine	
1.06	0.94	0.99	Cystine	
1.17	1.13	0.70	Glycerol	*Sugars and Sugar alcohols*
0.63	1.15	1.27	Glucose	
1.09	0.56	1.30	Sorbitol	
1.01	1.38	0.65	Sucrose	
1.00	1.29	0.74	Myristic acid	*Fatty acids and lipids*
1.06	1.12	0.83	Palmitic acid	
1.15	1.10	0.75	Linoleic acid	
1.10	1.15	0.76	Elaidic acid	
1.15	1.08	0.76	Arachidonic acid	
1.06	1.11	0.83	2-Monopalmitin	
1.11	0.93	0.95	Benzoic acid	*Others*
0.68	0.77	1.56	Salicylic acid	
0.43	0.57	2.01	Uric acid	
1.28	1.24	0.47	Inosine	
0.81	0.60	1.56	N.I. 1	

Figure S2. Heat map analysis for the relative abundance of different metabolites (VIP >1.0) derived from the GC-TOF-MS analysis. The colored squares (blue to red) indicate the fold changes normalized by the average of each metabolite. To determine the statistical significance, $p < 0.05$ obtained from one-way ANOVA was applied. Hp, *H. pylori*.
